# Two Cases of Inadequate Response to Remimazolam

**DOI:** 10.7759/cureus.42576

**Published:** 2023-07-27

**Authors:** Atsuhiro Kitaura, Shinichi Hamasaki, Hiroatsu Sakamoto, Shota Tsukimoto, Yasufumi Nakajima

**Affiliations:** 1 Anesthesiology, Kindai University Faculty of Medicine, Osaka, JPN

**Keywords:** tolerance, transcatheter aortic valve replacement, benzodiazepine analogues, bispectral index, benzodiazepine, anesthesia, remimazolam

## Abstract

We report the inadequate efficacy of remimazolam in two patients undergoing long-term benzodiazepine analog therapy. Remimazolam is a recently developed ultrashort-acting benzodiazepine. It is primarily used as an anesthetic in surgical procedures, as it has minimal effect on cardiac function and antagonists are available. It is expected to become more widely used in the future. On the other hand, similar to other benzodiazepines, benzodiazepine tolerance can also pose a challenge with remimazolam. Herein, we report two cases who were taking long-term oral benzodiazepine analogs. One patient did not fall asleep despite a sufficient dose of remimazolam and required a change to propofol. The other patient required a high dose of remimazolam to fall asleep; however, multiple signs of arousal were noted intraoperatively. Our findings suggest that remimazolam may not be an ideal anesthetic in long-term benzodiazepine analog users. Comprehensive assessment of preoperative medications and careful monitoring of intraoperative sedation levels are necessary. Furthermore, it may be advisable to consider the use of alternative agents such as propofol.

## Introduction

Remimazolam is a newly developed, ultra-short-acting benzodiazepine general anesthetic [1]. Remimazolam has a rapid onset of action and has the characteristic low cardiovascular impact of benzodiazepines [1-3]. It has been reported that it can be used safely in high-risk cases [3]. Remimazolam binds to the benzodiazepine receptor at the γ-aminobutyric acid sub-type A (GABAA) receptor, thereby potentiating the activity of γ-aminobutyric acid (GABA) and producing a sedative effect. Remimazolam is rapidly metabolized by carboxyelastase, and the degradation products are 300-400 times less active than the parent [4]. On the other hand, benzodiazepine tolerance is a complication of benzodiazepine usage; thus, it may also be a problem with remimazolam [5]. In addition, there is a risk of rapid accumulation of active metabolites. Herein, we report inadequate response to remimazolam in two cases undergoing long-term benzodiazepine analog therapy.

## Case presentation

Case 1

**Figure 1:**
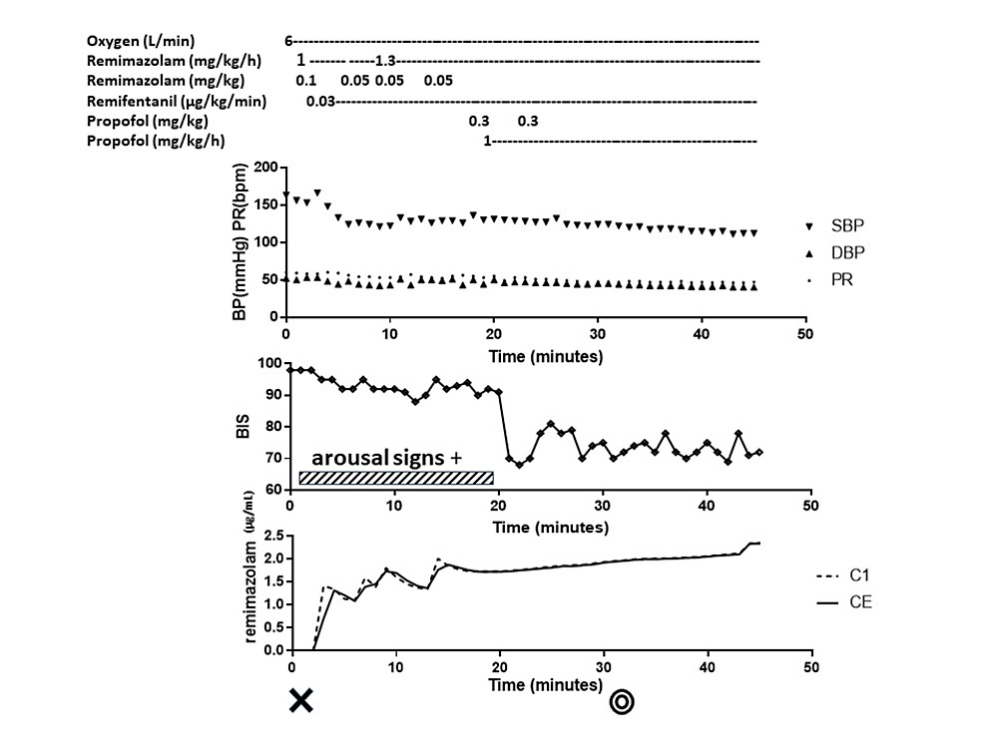
Anesthesia record of case 1 from anesthetic induction to the start of surgery Drugs administered, changes in hemodynamics, changes in BIS, and estimated concentration of remimazolam are shown. Hemodynamic parameters were stable. BIS remained in the 90s range after starting remimazolam administration. The patient remained aroused. Administration of propofol rapidly reduced the patient's level of consciousness and BIS. Predicted effect-site concentrations for remimazolam (Masui’s model) were consistently satisfactory. BP: blood pressure, SBP: systolic blood pressure, DBP: diastolic blood pressure, PR: pulse rate, BIS: bispectral index. C1: concentration of remimazolam in the first compartment (blood), CE: effect site concentration of remimazolam. Cross sign: the time to start the anesthesia. Double circle sign: the time to the start of the operation. The time on the horizontal axis is the elapsed time from the start of anesthesia in minutes. The shaded band represents the period of time during which the patient showed signs of arousal.

An 82-year-old man (height 173 cm, weight 79 kg) was referred to our hospital for severe aortic valve stenosis and was scheduled for transcatheter aortic valve replacement (TAVR). He had been regularly taking 0.5 mg of etizolam orally before bed every night for at least five years. The time of the last dose before the operation was 12 hours before the admission to the operating room. He was receiving hemodialysis due to diabetic nephropathy. He had hypertension and diabetes. Monitored anesthesia care (MAC) with remimazolam and remifentanil was planned. No premedication was administered. On arrival at the operation room, standard monitors for non-invasive blood pressure, continuous electrocardiography, pulse oximetry, and bispectral index (BIS; Aspect Medical Systems, Norwood, Massachusetts) were attached. Prior to the induction of anesthesia, an arterial pressure line was secured with local anesthesia. Remimazolam 0.1 mg/kg was administered, followed by 1 mg/kg/h. Three minutes after administration, the patient's eyes were still open, his consciousness was clear, and he was able to communicate orally. BIS was 96 and did not decrease. Therefore, remimazolam 0.05 mg/kg was added; however, the patient was still open-eyed and conscious, and his BIS remained unchanged. Remimazolam 0.05 mg/kg was further added; however, there was no change. On the third administration of remimazolam 0.05 mg/kg, BIS was 93, which was not considered a sufficient decrease; thus, we stopped administering anesthesia with remimazolam and started propofol 0.3 mg/kg. The patient promptly became unconscious, and BIS decreased to 60. His anesthesia was managed with propofol and dexmedetomidine thereafter, and TAVR was completed. The patient awoke spontaneously and promptly after the completion of the TAVR procedure. Flumazenil was not administered because one hour had passed since the end of remimazolam administration, and the patient was well awake. He had no memory of the induction of anesthesia. His anesthesia records are shown in Figure 1.

Case 2

**Figure 2:**
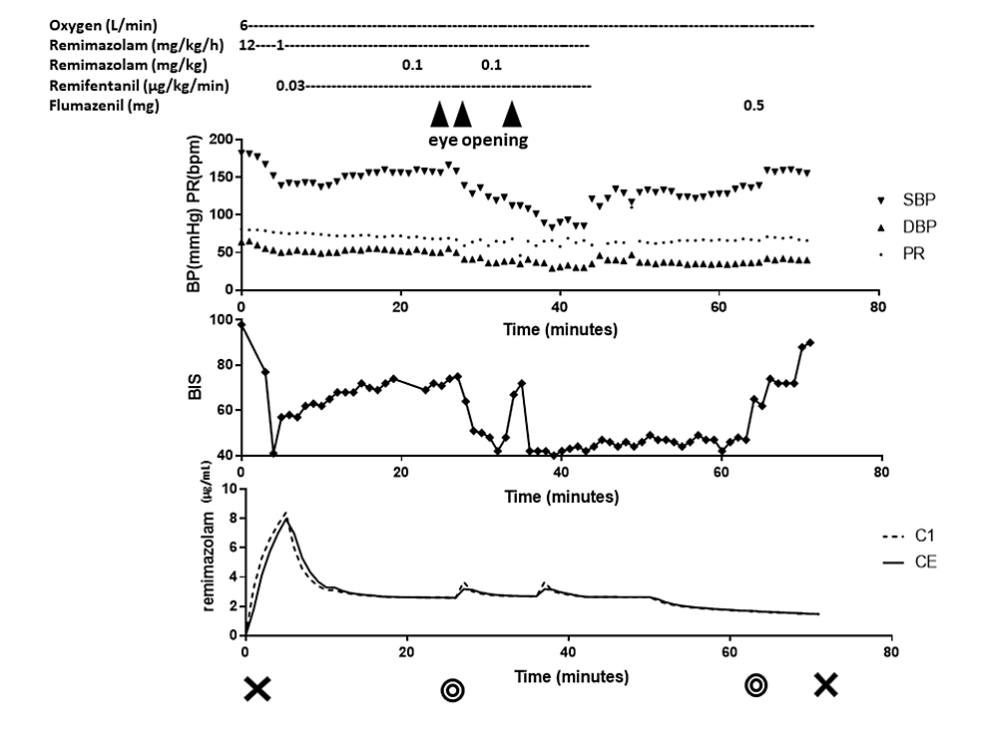
Anesthesia record of case 2 Drugs administered, changes in hemodynamics, changes in BIS, and estimated concentration of remimazolam are shown. BIS rapidly reduced after induction and each additional bolus of remimazolam. BIS was also transiently elevated when the patient showed signs of alertness. The patient remained aroused. Administration of propofol rapidly reduced the patient's level of consciousness and BIS. Predicted effect-site concentrations for remimazolam (Masui’s model) were consistently satisfactory. BP: blood pressure, SBP: systolic blood pressure, DBP: diastolic blood pressure, PR: pulse rate, BIS: bispectral index. C1: concentration of remimazolam in the first compartment (blood), CE: effect site concentration of remimazolam. Cross sign: the start and end of anesthesia. Double circle sign: the start and end of the operation. The time on the horizontal axis is the elapsed time from the start of anesthesia in minutes. The triangle indicates the point at which the patient showed signs of arousal.

An 89-year-old female (height 143 cm, weight 45 kg) was scheduled for TAVR for severe aortic stenosis. She had a medical history of moderate mitral regurgitation and poor renal function (creatinine: 1.42 mg/dL). In the operating room, anesthesia was induced with remimazolam 12 mg/kg/h after basic monitoring, and an arterial pressure line was secured. The patient required five minutes to fall asleep, and BIS decreased to 57. Intraoperatively, anesthesia was maintained with MAC using remimazolam 1 mg/kg/h and remifentanil 0.03 ug/kg/min. Intraoperatively, 1% lidocaine was used as local anesthesia during invasive procedures such as vascular puncture and sheath insertion. However, during skin disinfection, femoral artery puncture, and sheath insertion, the patient opened her eyes and clearly complained of pain. She was mobile and able to perform indicated movements, and her BIS increased to the high 70s. A bolus dose of remimazolam 0.1 mg/kg was administered at each sign of arousal and anesthesia was maintained with remimazolam and remifentanil until the end. Her anesthesia records are shown in Figure 2. After the surgery, she awoke spontaneously. Because the remimazolam dose was high, 0.5 mg flumazenil was administered. When the patient awoke, we found that she had no intraoperative memory. Fortunately, there was no withdrawal symptom. A careful postoperative history revealed that the patient had been taking brotizolam 0.25 mg orally as a sleep aid for insomnia for at least three years. The time of the last dose before the operation was 15 hours before admission to the operating room.

## Discussion

We experienced two cases of inadequate response to remimazolam in patients who were taking benzodiazepine analogs or their derivatives on a long-term basis. In case 1, etizolam had been used for more than five years. In this patient, remimazolam 0.25 mg/kg bolus, 1 mg/kg/h continuous dose, and remifentanil 0.03 µg/kg/min did not help the patient fall asleep. However, when we switched to propofol, the patient fell asleep quickly at 0.3 mg/kg. Case 2 had been on brotizolam for more than three years. This patient was also given remimazolam at 12 mg/kg/h but required five minutes to become unconscious. The dose of remimazolam needed for induction was 1 mg/kg. Intraoperatively, a continuous dose of remimazolam 1 mg/kg/h and remifentanil 0.03 mg/kg/min was insufficient to prevent signs of arousal.

In a previous phase I trial, remimazolam put all healthy adults to sleep at 0.2 mg/kg and all elderly patients at 0.1 mg/kg [6]. In phase IIb/III trials, all patients were put to sleep at doses ranging from 0.04 to 0.275 mg/kg [7]. The effect-site concentration of remimazolam required for general anesthesia is estimated to be 0.6 to 1 µg/mL [8]. According to Masui's pharmacokinetic model [8], sufficient effective site concentrations were achieved in both the present cases (Figures 1, 2). Considering that the patients were elderly and taking benzodiazepine analogs, a reasonable dose of remimazolam was administered to make them fall asleep. Therefore, our findings suggest that remimazolam was not sufficiently effective in these cases.

In the present report, we used BIS as an indicator of sedation in addition to the patient's signs of arousal. However, the BIS algorithm was created for different drugs; thus, it may not be a suitable measure of sedation for benzodiazepines [9,10]. In a phase I study, remimazolam caused 95% and 50% of subjects to lose consciousness, with BIS values of 54 and 66, respectively, suggesting that BIS can be used to assess the state of consciousness [6]. To some extent, BIS and patient arousal were correlated in both of the present patients. BIS values indicated that remimazolam was inadequately effective in Case 1, as BIS did not decrease even after a large single dose. In Case 2, BIS decreased immediately after induction and after additional bolus and gradually increased during continuous dosing. Thus, it was considered that the required dose was higher than expected but did have an effect.

Long-term use of benzodiazepines is known to result in benzodiazepine tolerance. Several mechanisms of benzodiazepine tolerance have been reported [11]. In Case 1, propofol, which binds to multiple subunits of GABA and sites other than benzodiazepine receptors, was effective. Thus, possible mechanisms include reduced allosteric uncoupling of GABAA and benzodiazepine binding sites [12] and modulation of GABAA receptor subunit composition [11]. Remimazolam cross-tolerance is still unclear, and further studies are warranted.

There have been several reports of remimazolam tolerance in regular benzodiazepine users [13-15]; however, in these previous studies, it was unclear how long patients had been using benzodiazepines and their analogs on a regular basis to develop tolerance. The degree of tolerance also varies widely among patients, ranging from complete ineffectiveness to an increase in the required dose. In addition, the effect of benzodiazepines is reported to vary widely from individual to individual [16]. Therefore, when using remimazolam, examination of preoperative medications and careful monitoring of intraoperative sedation levels are important. For long-term benzodiazepine analog users, it may be advisable to use alternative agents such as propofol.

## Conclusions

Remimazolam may not be an ideal anesthetic for patients who are on long-term medication with drugs derived from benzodiazepine analogs. When anesthetizing patients using remimazolam, examination of preoperative medications and careful monitoring of intraoperative sedation levels are necessary.
